# The usefulness of C-reactive protein and neutrophil-to-lymphocyte ratio for predicting the outcome in hospitalized patients with liver cirrhosis

**DOI:** 10.1186/s12876-015-0378-z

**Published:** 2015-10-23

**Authors:** Jung Hyun Kwon, Jeong Won Jang, Young Woon Kim, Sung Won Lee, Soon Woo Nam, Dongwook Jaegal, Seungok Lee, Si Hyun Bae

**Affiliations:** 1 Department of Internal Medicine, Incheon St. Mary’s Hospital, The Catholic University of Korea, 56 Dongsu-ro, Bupyeong-gu, Incheon South Korea; 2 Department of Internal Medicine, Seoul St. Mary’s Hospital, The Catholic University of Korea, 222 Banpo-daero, Seocho-gu, Seoul South Korea; 3 Department of Internal Medicine, Bucheon St. Mary’s Hospital, The Catholic University of Korea, 327 Sosa-ro, Wonmi-gu, Bucheon-si, Gyeonggi-do South Korea; 4 Department of Laboratory Medicine, Incheon St. Mary’s Hospital, The Catholic University of Korea, 56 Dongsu-ro, Bupyeong-gu, Incheon South Korea

**Keywords:** C-reactive protein, Liver cirrhosis, Infection, Neutrophil-to-lymphocyte ratio, Survival

## Abstract

**Background:**

The role of clinical parameters such as systemic inflammatory response syndrome (SIRS) criteria in predicting the infection remains unclear in cirrhosis patients. The aim was to evaluate the usefulness of inflammatory markers including C-reactive protein (CRP) and the neutrophil-to-lymphocyte ratio (NLR) for diagnosis of infection and predicting the outcomes in hospitalized cirrhotic patients.

**Methods:**

The study included 184 cirrhotic patients consecutively hospitalized from 2011 to 2012. The presence of overt infection and survival was evaluated. CRP concentration, NLR, Model for End-Stage Liver Disease (MELD) score and the presence of SIRS were assessed.

**Results:**

The main cause of admission was uncontrolled ascites (36.4 %), followed by varix bleeding (23.9 %), and hepatic encephalopathy (13.6 %). Fifty-eight patients (31.5 %) had overt infection during hospitalization and thirty-two patients (17.4 %) expired during the follow up period (median 38 months). Ninety-two patients (52.2 %) fulfilled the SIRS criteria and among them, only 32 patients (38.5 %) had the overt infection. For diagnose of the infection, baseline CRP concentration was a significant factor compared to the presence of SIRS (odds ratio 1.202, *P* = 0.003). For predicting one-month short-term survival, MELD score, NLR and WBC count were significant factors but in Child-Pugh class C patients, NLR was only an independent factor.

**Conclusions:**

CRP was a significant indicator of infection in hospitalized cirrhotic patients and a NLR was a useful predictor of 1-month survival, particularly in Child–Pugh class C patients. This study suggests that the inflammatory markers such as CRP and NLR can help identify cirrhotic patients at risk of unfavorable outcomes.

## Background

In cirrhotic patients, the severity of liver disease is an important factor influencing the risk of mortality [1, 2]. Systemic inflammation occurs frequently in patients with advanced cirrhosis [2, 3] and might be associated with a negative outcome [4]. Bacterial infections can lead to both a longer hospital stay and increased mortality in patients with cirrhosis [5–7]. Previous studies have reported that bacterial infections are present or develop during hospitalization in around the one-third of hospitalized cirrhotic patients [3, 6, 8]. Systemic inflammatory response syndrome (SIRS) has been strongly suggestive of the presence of infection and association with poor outcomes [2, 4, 9]. However, the specific characteristics of cirrhotic populations, such as hypersplenism, hyperventilation associated with hepatic encephalopathy and hepatorenal syndrome, increase the difficulty of identifying episodes and the use of beta-blockers may modify the clinical and biochemical parameters used to identify SIRS [10]. Thus, the presence of overt infection cannot be excluded in patients with cirrhosis who do not meet the SIRS criteria. Although the liver function markers such as the Model for End-Stage Liver Disease (MELD) score is a good factor for survival, it has some limitations to reflect the acute status of hospitalized cirrhotic patients. Hospitalization in cirrhotic patients would be required at the decompensated events or infection and so on. This is why a convenient and easy-to-measure parameter is needed for predicting the outcomes in the unstable patients group.

C-reactive protein (CRP), which is synthesized in the liver, has been identified as an inflammatory marker [11]. We previously reported the usefulness of CRP in predicting short-term mortality in patients with hepatocellular carcinoma (HCC), and outcomes after liver transplantation [12, 13]. CRP is also an acute phase reactant that is suggested to be a marker of the presence of bacterial infection [14]. The neutrophil-to-lymphocyte ratio (NLR) has been suggested as a marker of systemic inflammation and shows the relationship between two different immune pathways [15]. The neutrophil count reflects ongoing inflammation, whereas the lymphocyte count represents the immune regulatory pathway. The NLR has been used to predict outcomes in patients with cancer and cardiac disease [15, 16]. It was recently reported to predict the outcomes in patients with HCC, nonalcoholic fatty liver disease, and liver transplantation [12, 17, 18], Although clinical risk factors of bacterial infection, such as advanced liver diseases or gastrointestinal bleeding, are well characterized [6, 8, 19–21], data on both CRP and NLR as serologic markers are scarce in such patients with advanced diseases. The aim of this study was to investigate whether CRP and NLR could significantly improve the diagnostic accuracy of infection and advance the predictive power of short-term mortality in a large consecutive cohort of patients, who were hospitalized due to hepatic events.

## Methods

### Patients

This was a retrospective observational cohort study performed at a university hospital. The study included a cohort of 184 consecutive cirrhotic patients hospitalized in a tertiary center from September 2011 to September 2012. The diagnosis of cirrhosis was based on liver biopsy or clinically when the patients had at least two of the following three criteria: an inhomogeneous hepatic surface with splenomegaly or portal hypertension on radiological findings; platelet count <100,000/mm3 or variceal changes on endoscopy [22]. The exclusion criteria were a previous diagnosis of HCC or extrahepatic malignancy and an elective admission to evaluate the suspicion of HCC. This study was approved by the Office of Human Research Protection Program, Catholic Medical Centre (CMC OHRP, reference number; OC13RISI0127) and the need for consent from participants was waived by the CMC OHRP. This study was also in accordance with the Helsinki Declaration of 1975.

The main clinical events for hospitalization such as uncontrolled ascites, variceal bleeding, hepatic encephalopathy, or other signs of hepatic deterioration were reviewed carefully and evaluated. When there was more than one main clinical event at admission, the criteria used to define the main cause of admission were the following: (a) Whenever variceal bleeding was coincident with other major complications, it was considered the main cause of admission, (b) In patients with hepatic encephalopathy and other events, the former was considered the main cause of admission, (c) All the types of infection were counted and analyzed even if it was a single present or co-exist with other clinical events [3].

At admission, patients routinely underwent a physical examination, laboratory tests, and X-ray of the chest and abdomen. Laboratory tests included blood chemistry and blood cell counts including CRP concentration and ascitic/pleural fluid cell counts. Serum CRP level was measured as high-sensitivity CRP by an immunoturbidimetric assay using the C-Reactive Protein, High Sensitivity reagent (Beckman Coulter, Inc., Fullerton, CA, USA; limit of detection, 0.08 mg/L). The NLR was calculated by dividing the neutrophil count by the lymphocyte count. All the hospitalized patients were administered empiric broad-spectrum antibiotics (ceftriaxone or ciprofloxacin) after initial laboratory test and physical examination, and the regimen was modified or stopped according to the results of the cultures or the infection [7].

During hospitalization, cultures of blood, urine, ascites, pleural effusion, and sputum or swabs were taken when an infection was suspected. The severity of liver disease was assessed according to the Child–Pugh class and the MELD score. Physical examination and laboratory tests to measure blood cell counts and chemistry were daily performed. Depending on the creatinine level, abdominal ultrasonography or computed tomography was performed if needed.

### Definition of systemic inflammation and infection

SIRS was assessed according to the recommendations of the American College of Chest Physicians/Society of Critical Care Medicine Consensus Conference [23]. Patients were considered to have SIRS if they fulfilled at least two of the following criteria: (a) core temperature of >38 °C or <36 °C; (b) heart rate of >90 beats/min; (c) respiratory rate of >20 breaths/min; partial carbon monoxide pressure (PaCO2) ≤ 32 mmHg or the need of mechanical ventilation or (d) white blood cell (WBC) count of >12,000/mm^3^ or <4000/mm^3^, or differential count showing >10 % immature polymorphonuclear neutrophil cells (PMNCs).

The most common bacterial infection in cirrhosis is known to be spontaneous bacterial peritonitis (SBP), urinary tract infection, followed in frequency by pneumonia, and bacteremia [3, 6, 24]. For this evidence, diagnosis of SBP was considered as an ascitic fluid PMNC count >250/mm^3^. Urinary tract infection was diagnosed as a urinary WBC count >10 cells per high-power field or a positive urine culture [3]. Pneumonia was diagnosed if there was radiographic evidence of pulmonary infiltration associated with purulent sputum. Bacteremia was diagnosed as a positive blood culture in the absence of any recognized source of infection [3, 4]. Other infections such as cellulitis, biliary infection, and enterocolitis were diagnosed according to clinical, radiological, and bacteriologic data. Community-acquired infections were defined as those that were recognized before admission or within the first 48 h, and hospital-acquired infections or delayed infections were diagnosed after this period [3].

### Statistical analysis

SPSS version 18 (SPSS Inc., Chicago, IL, USA) was used to analyze the data. The data was expressed as the mean ± SD or as the median and range. Logistic regression analysis was performed to assess significant differences in predicting 1-month survival and the presence of infection. Overall survival was calculated from the date of admission to the date of death or the last follow-up. For patients undergoing liver transplantation, follow-up was censored at the time of transplantation. The factors affecting the survival rate were identified using Cox’s proportional-hazard model.

## Results

### Baseline characteristics of hospitalized cirrhotic patients with and without infection

One hundred eighty-four patients were included in the study (Table 1): 125 (67.9 %) men with a mean age of 56.7 ± 11.7 years. The main clinical events for hospitalization were uncontrolled ascites in 67 patients (36.4 %), variceal bleeding in 44 patients (23.9 %), and hepatic encephalopathy in 25 patients (13.6 %), alcoholic withdrawal seizure in 6 patients (3.2 %). Most patients had severe liver disease of Child–Pugh class B or C (82.5 %) and average MELD score was 12.2 ± 7.5.

**Table 1:** Baseline characteristics of hospitalized cirrhotic patients with or without infection

	Patients with infection	Patients without infection	*P* values
	(*n* = 58, 31.5 %)	(*n* = 126, 68.5 %)	
Age (year-old)	60.2 ± 12.4	55.0 ± 11.0	0.005
Sex (male, %)	33 (56.9 %)	92 (73 %)	0.030
HBV/HCV/Alc/others	20/3/27/8	31/11/82/2	0.002
Child–Pugh classification (A/B/C)	11/26/21	23/59/44	0.969
MELD score	13.2 ± 8.0	11.9 ± 19.1	0.215
Meet the criteria of SIRS (%)	37 (63.8 %)	59 (46.9 %)	0.032
Body temperature	36.8 ± 0.9	36.3 ± 0.5	0.000
Neutrophil to lymphocyte ratio	8.3 ± 10.1	4.9 ± 6.8	0.027
WBC count (/mm^3^)	8971.7 ± 5340.8	7362.5 ± 4668.9	0.100
Neutrophil count (% of WBC)	71.8 ± 13.7	65.1 ± 14.0	0.003
Lymphocyte count (% of WBC)	17.8 ± 10.9	23.0 ± 12.1	0.004
CRP (mg/L)	34.1 ± 49.2	11.9 ± 19.1	0.002
Baseline laboratory data			
Platelet count (/mm^3^)	116.8 ± 70.0	105.2 ± 55.3	0.272
Albumin (g/dL)	3.0 ± 0.7	3.0 ± 0.6	0.794
Total bilirubin (mg/dL)	3.3 ± 3.1	3.9 ± 4.6	0.373
Prothrombin time (INR)	1.5 ± 0.6	1.5 ± 0.5	0.937
Creatinine (mg/dL)	1.1 ± 0.9	0.9 ± 0.7	0.118
Na (mEq/L)	132.2 ± 18.3	134.9 ± 12.5	0.223

*MELD* model for end-stage liver disease, *SIRS* systemic inflammatory response syndrome, *CRP* C-reactive protein, *INR* international normalized ratio

Differences assessed using the Mann–Whitney *U* test for numerical variables and chi-test for categorical variables

Fifty-eight of 184 patients (31.5 %) had overt infection during admission, of whom 49 (85 %) patients were diagnosed a community acquired infection. The most common infections were urinary tract infection (*n* = 21, 36.2 %) followed by pneumonia/bacteremia (*n* = 9/9, 15.5/15.5 %), SBP (*n* = 6, 10.3 %), cellulitis/biliary tract infection/enterocolitis (*n* = 3/3/3, 5.2/5.2/5.2 %) and others (*n* = 4, 6.9 %). The patients with infection were old and female dominant compared to those without infection. However, the alcohol related cirrhosis in the patients with infection was less than that in the patients without infection although it was the most common cause of underlying liver cirrhosis (Table 1). The number of patients who met the SIRS criteria at admission was 96 (52.2 %). The patients with infection fulfilled the SIRS criteria significantly more than those without infection (63.8 % *versus* 46.9 %, *P* = 0.032). Among the SIRS criteria, the body temperature in patients with infection was significantly higher than those without infection. Otherwise, heart rate, respiration rate and PaCO_2_ levels did not differ between patients with and without infection. The platelet counts and any other liver panel tests were not different depending on the presence of infection.

### Baseline CRP level predicts the infection in hospitalized cirrhotic patients

**Fig. 1:**
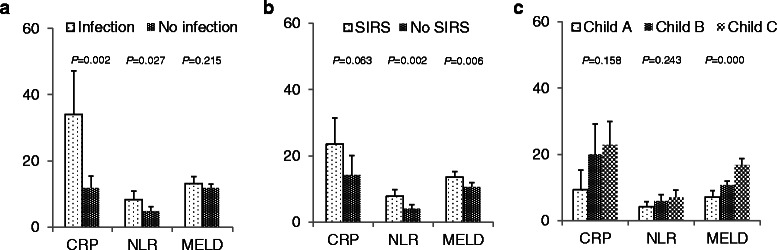
**a** CRP level and NLR were significantly higher in infected patients than uninfected patients. **b** NLR and MELD score in patients with SIRS were significantly higher than in those without SIRS. **c** CRP level, NLR and MELD score increased with progressing Child-Pugh class, but MELD score only reached the statistical difference. Boxes and bars show the means and 95 % confidence intervals. CRP, C-reactive protein; NLR, neutrophil to lymphocyte ratio; MELD, model for end-stage liver disease; SIRS, systemic inflammatory response syndrome

The levels of CRP in patients with infection were significantly higher than those without infection (Fig. 1a). In addition, NLR was significantly higher in patients with infection than those without, in whom WBC counts were not different between the two groups (Fig. 1a). Specifically, the neutrophil count was higher and the lymphocyte count was lower in cirrhotic patients with infection than those without (Table 1). However, the MELD score did not differ depending on infection. Nine out of 45 patients developed delayed infection after admission, whose CRP and NLR did not differ compared to those with community-acquired infection.

For predicting infection using the logistic regression analysis, old age, female gender, non-alcohol related liver cirrhosis, the presence of SIRS, high level of CRP and NLR were significant factors (Table 2). In the multivariate analysis, female gender and high baseline CRP were predictable risk factors for the infection in hospitalized cirrhotic patients.

**Table 2:** Logistic regression analysis for predicting the infection in hospitalized cirrhotic patients

	Logistic regression analysis			
Baseline variables	Univariate analysis	Multivariate analysis		
	*P* values	*P* values	Odds ratio	95 % CIs
Age	0.006	0.120	1.024	0.994-1.056
Female	0.031	0.042	2.284	1.030-5.065
Non-alcoholic LC	0.018	0.162	1.727	0.803-3.717
Child-Pugh score	0.976			
MELD score	0.216			
SIRS	0.034	0.316	1.454	0.700-1.034
CRP	0.001	0.003	1.202	1.007-1.304
NLR	0.019	0.181	1.034	0.985-1.086
WBC	0.086			

*CI* confidence intervals, *LC* liver cirrhosis, *MELD* model for end-stage liver disease, *SIRS* systemic inflammatory response syndrome, *CRP* C-reactive protein, *NLR* neutrophil-to-lymphocyte ratio

With regard to SIRS, CRP showed the trend to increase in patients showing the SIRS (Fig. 1b). NLR and MELD score in the patients who met the SIRS criteria were significantly higher than those who did not (Fig. 1b). For our cohort, the level of the CRP and the NLR positively correlated with the Child-Pugh class but it didn’t show the statistical significance (Fig. 1c).

### Neutrophil to lymphocyte ratio predicts the short-term survival in hospitalized cirrhotic patients

Thirty-two patients expired during the mean follow up period 429.3 ± 302.4 days, of whom eight patients expired within one month of admission. Patients with high MELD had significantly poor survival regardless of the presence of infection or SIRS (*P* = 0.000). However, for predicting one-month survival after the hospitalization, the baseline NLR as well as MELD score and WBC count was a significant factor (Table 3). Especially, in the patients with Child-Pugh class C, a high NLR was an independent predictor for the one-month survival after admission (Table 3).

**Table 3:** Logistic regression analysis for predicting the one-month short survival in the hospitalized cirrhotic patients

	Univariate analysis	Multivariate analysis					
Baseline variables	Total patients	Child–Pugh A and B patients			Child–Pugh C patients		
		(*n* = 119)			(*n* = 65)		
	*P* values	*P* values	OR	95 % CIs	*P* values	OR	95 % CIs
Age	0.571						
Female	0.230						
Alcoholic LC	0.893						
MELD score	0.001	0.161	0.610	0.828-3.131	0.159	1.130	0.954-1.338
CRP	0.122						
NLR	0.005	0.269	0.446	0.106-1.866	0.022	1.246	1.032-1.505
WBC	0.011	0.169	1.001	1.000-1.001	0.496	1.000	1.000-1.000
SIRS	0.997						
Infection	0.657						

*OR* odds ratio, *CI* confidence intervals, *LC* liver cirrhosis, *MELD* model for end-stage liver disease, *CRP* C-reactive protein, *NLR* neutrophil-to-lymphocyte ratio, *SIRS* systemic inflammatory response syndrome

## Discussion

The hospitalized cirrhotic patients suffer from acute decompensated events underlying chronic inflammation status. The present study revealed that CRP and NLR are useful diagnostic markers for infection compared to the SIRS in hospitalized cirrhosis patients. In addition, the baseline NLR predicted the one-month survival as did the MELD score in hospitalized cirrhosis patients especially with Child–Pugh class C.

As expected, 82.5 % of the patients in this study had severe liver disease with Child-Pugh class B or C, and over half of patients (52.2 %) had SIRS at inclusion. We know that the patients with advanced cirrhosis have a spontaneous increased proinflammatory response compared with noncirrhotic patients because of an imbalance between proinflammatory (enhanced) and anti-inflammatory (inhibited) signaling pathways in immune cells [2, 25, 26]. However, the specific characteristics of cirrhotic patients increase the difficulty of identifying SIRS and the presence of SIRS itself do not directly diagnose the infection. Infection was a severe but frequent (20-60 %) complication with decompensated cirrhosis although frequently asymptomatic, and accounted for increased mortality [3, 6]. In present study, 46.8 % of patients without infection also had SIRS although the patients with infection had more SIRS than those without infection. In a previous study [27], 46 % of infected patients with cirrhosis did not have SIRS. Taken together, not all infected patients with cirrhosis develop SIRS. The authors focused on new prognostic factors that could reveal acute status such as infection in addition to standard MELD score and SIRS.

In the present study, CRP level was an independent predictor of infection compared to the SIRS and MELD scores. CRP is an acute-phase reactant synthesized by hepatocytes in response to inflammation and regulated by proinflammatory cytokines [28]. In our previous report [29], CRP level was an important factor for survival, and correlated with unfavorable tumor characteristics of HCC. The merit of CRP concentration is a simple marker that can be frequently checked in admitted patients. In another reports, CRP levels were higher in patients with SIRS, infection, or alcoholic hepatitis [8, 14, 30] and CRP was the most useful diagnostic marker of infection compared with other acute phase reactants such as the procalcitonin, lipopolysaccharide-binding protein, sCD14 [14]. In addition, CRP level was reported associated with a lower response rate to antibiotics, a higher mortality rate in cirrhosis patients with SBP [31]. The present study consists that CRP levels increased in patients with infection, SIRS and worse liver function but the most distinguishable increase was shown in patients with infection. Taken together, our study and previous studies suggest that the CRP may be a surrogate marker for the early identification of infection in hospitalized cirrhotic patients [8, 14, 30, 31].

NLR increased in patients with infection and predicted the short-term outcome in hospitalized cirrhotic patients. An increased WBC count might mean the presence of infection, but cirrhotic patients usually have low WBC counts due to hypersplenism and patients with alcoholic cirrhosis show high WBC count even without infection. In the present study, the simple WBC counts were not different between infection and no infection group. However, neutrophil count in the infection group was higher than no infection group and lymphocyte count in the infection group was lower than no infection group. This suggests that NLR is a more powerful predictor of infection than simple WBC counts. The early hyperdynamic phase of infection is characterized by a proinflammatory state, which is associated with suppression of neutrophil apoptosis [32] and increased lymphocyte apoptosis in the thymus and spleen [33]. Recently, the neutrophil dysfunction was reported even in the stable cirrhosis patients [34] and predicted outcomes in cirrhosis and alcohol hepatitis [35]. It might be due to the deranged phagocytic activity of opsonized bacteria of neutrophil in advanced cirrhosis. In the present study, neutrophil also showed the significant difference between infection and no infection group. Until now, there is little about the diagnostic power of NLR for infection in liver cirrhosis even though there are some reports about the role of NLR in infection among patients with acute exacerbation of chronic obstructive pulmonary disease and in emergency department [36, 37]. Therefore, NLR can be a helpful marker for identifying infection in hospitalized cirrhotic patients in addition to CRP.

Accumulating data have shown that NLR may be the outcome predictor of several cancers, cardiac disease and even in liver disease such as HCC, non-alcoholic steatohepatitis, liver transplantation waiting patients [12, 15–18]. Recently, pretreatment NLR was reported to be associated with the prognosis of patients with acute on chronic hepatitis B liver failure [38]. However, there has been not yet clear explanation how elevated NLR would be responsible for liver cirrhosis patients. As previously mentioned, in cirrhotic patients, immune and inflammatory systems are activated and inflammatory markers, such as interleukin-6 and tumor necrosis factor-α, have been found to be elevated [39]. Since neutrophil could also suppress T cell activation through the production of arginase, nitric oxide and reactive oxygen species [40], it induces depletion of lymphocyte-mediated immune response. Therefore, elevated NLR might be a poor prognostic marker in cirrhosis patients. In a previous report in which the stable cirrhosis patients without infection or HCC were enrolled, the NLR predicted survival at 12, 24, and 36 months [41]. By contrast, our patients mostly showed decompensation at admission and NLR could predict one-month survival especially in patients with Child-Pugh class C. The overall survival of cirrhotic patients is influenced by many factors such as liver function and the presence of life-threatening episodes. Therefore, the MELD and Child–Pugh scores could predict the overall survival independent of the presence of infection. The NLR reflects the interrelationship between the lymphomononuclear and neutrophilic arms of the process of inflammation through complex cytokine interactions [41]. Thus, the NLR is an indicator of the overall inflammatory status of the body. The NLR was a better predictor of 1-month survival than 3- or 6-month survival (data not shown). These findings suggest that NLR reflects the current inflammatory status at admission and thus may be useful for predicting short-term survival for 1 month even in Child–Pugh class C patients whose liver function is poor. In our previous report, NLR was significantly related to underlying hepatic reserve as well as tumor burden [12]. Thus, NLR might be helpful to consider who need the early liver transplantation in addition MELD score.

## Conclusions

In conclusion, CRP and NLR are helpful diagnostic markers of infection in hospitalized cirrhotic patients. In Child–Pugh class C patients, an elevated NLR predicted a poor 1-month survival. Therefore, hospitalized cirrhotic patients with an elevated CRP concentration and NLR should be monitored carefully for the presence of infection. In addition to the classical MELD score, NLR may be a useful predictor of the short-term mortality in hospitalized cirrhotic patients.

## Abbreviations

SIRS: Systemic inflammatory response syndrome
MELD: Model for End-Stage Liver Disease
CRP: C-reactive protein
HCC: Hepatocellular carcinoma
NLR: Neutrophil-to-lymphocyte ratio
PaCO2: Partial carbon monoxide pressure
WBC: White blood cell
PMNC: Polymorphonuclear neutrophil cell
SBP: Spontaneous bacterial peritonitis
